# Population Pharmacokinetics and Antimalarial Pharmacodynamics of Piperaquine in Patients With *Plasmodium vivax* Malaria in Thailand

**DOI:** 10.1038/psp.2014.29

**Published:** 2014-08-27

**Authors:** J Tarning, P Thana, A P Phyo, K M Lwin, W Hanpithakpong, E A Ashley, N P J Day, F Nosten, N J White

**Affiliations:** 1 Mahidol-Oxford Tropical Medicine Research Unit, Faculty of Tropical Medicine, Mahidol University, Bangkok, Thailand; 2 Centre for Tropical Medicine, Nuffield Department of Medicine, University of Oxford, Oxford, UK; 3 Shoklo Malaria Research Unit, Mahidol-Oxford Tropical Medicine Research Unit, Faculty of Tropical Medicine, Mahidol University, Tak, Thailand

## Abstract

Dihydroartemisinin-piperaquine is an effective drug in the treatment of *Plasmodium falciparum* and *P. vivax* malaria. The objective of this study was to evaluate the population pharmacokinetics and pharmacodynamics of piperaquine in patients with *P. vivax* malaria in Thailand after a standard regimen of dihydroartemisinin-piperaquine to determine whether residual piperaquine prevents or delays the emergence of *P. vivax* relapse. Sparse blood samples were collected from 116 patients. Piperaquine pharmacokinetics were described well by a three-compartment distribution model. Relapsing *P. vivax* malaria was accommodated by a constant baseline hazard (8.94 relapses/year) with the addition of a surge function in a fixed 3-week interval and a protective piperaquine effect. The results suggest that a large proportion of the first relapses were suppressed completely by residual piperaquine concentrations and that recurrences resulted mainly from emergence of the second or third relapse or from reinfection. This suggests a significant reduction in *P. vivax* morbidity when using dihydroartemisinin-piperaquine compared with other antimalarial drugs with shorter terminal postprophylactic effects.

*Plasmodium vivax* is the most difficult of the five species of human malaria parasites to eliminate. It is the most geographically widespread human malaria parasite, causing an estimated 130–390 million cases every year.^1^ *P. vivax* is transmitted within 95 countries in America, Africa, and Asia.^2,3^ In total, 2.85 billion people were estimated to be at risk of *P. vivax* transmission in 2009, especially in Central and Southeast Asia, where more than 90% of the world's *P. vivax* transmission occurs.^3^ Acute *P. vivax* malaria is rarely life threatening, but it causes substantial morbidity in higher transmission areas as a result of multiple relapses from latent liver-stage parasites.^4^ These repeated infections may result in life-threateningly severe anemia due to destruction of both infected and noninfected red blood cells. *P. vivax* is an important cause of low birth weight.^4,5,6,7^

Chloroquine, a blood schizonticide, is used in first-line treatment and prophylaxis of *P. vivax* malaria in most endemic areas. However, *P. vivax* resistance to chloroquine, which was first observed in Papua New Guinea in 1989,^8^ is increasing in many parts of the world.^9^ A combination of two drugs with different mechanisms of action is preferred for the treatment of malaria to reduce the risk of the development of resistant parasite strains. The fixed combination of dihydroartemisinin-piperaquine (DHA-PQ) is recommended by the World Health Organization as the first-line treatment of uncomplicated *P. falciparum* malaria^10^ and has replaced chloroquine in the first-line treatment of resistant *P. vivax* malaria in Indonesia. DHA-PQ is currently available as a fixed-dose combination, administered once daily over 3 days. A meta-analysis pooled from 12 different studies in 6 countries between 2003 and 2006 showed high efficacy against *P. falciparum* malaria (polymerase chain reaction-corrected cure rates of 98.7% at day 28) and good tolerance (4.8% total incidence of early vomiting) of DHA-PQ.^11^ DHA-PQ also has excellent efficacy against *P. vivax* malaria.^12,13,14^

Piperaquine is distributed extensively into tissues, resulting in multiphasic distribution and a slow elimination from the systemic circulation (elimination half-life (*t*_1/2_) of ~18–28 days). Similar pharmacokinetic properties have been reported in children but these pediatric studies suggest that small children need a higher weight-based dose to achieve exposure comparable to that in older children.^15^ Most piperaquine pharmacokinetic studies have sampled venous plasma. However, capillary sampling is a desirable alternative for field pharmacokinetic studies, especially in children. The population pharmacokinetics and pharmacodynamics of piperaquine have not been reported previously in patients with *P. vivax* malaria.

In South East Asia and Oceania, *P. vivax* relapses at 3-week intervals. Treatment with efficacious slowly eliminated antimalarials results in substantially delayed emergence of the relapse, but it has been unclear whether the first relapse is prevented (and so the second relapse is observed) or delayed. The aim of this study was to characterize the population pharmacokinetics and antimalarial pharmacodynamics of piperaquine in the treatment of *P. vivax* malaria and to use this information to determine whether piperaquine prevents or delays the first *P. vivax* relapse.

## Results

### Safety and efficacy

This study was conducted at Shoklo Malaria Research Unit clinics, Mae Sot, Thailand, an area of low and seasonal malaria transmission located at the Thailand–Myanmar border. This pharmacokinetic study was nested into a larger efficacy trial in 500 patients with *P. vivax* malaria infections. Full clinical details have been published elsewhere and DHA-PQ was found to be an effective alternative treatment with a lower cumulative risk of recurrent *P. vivax* malaria at 9 weeks compared with chloroquine (54.9 vs. 79.1%).^16^ Two-hundred and fifty Karen and Burmese patients with *P. vivax* malaria infections were enrolled in the DHA-PQ-treated arm of the efficacy study (**Table 1**). All patients received DHA-PQ (Duo-cotecxin, Beijing Holley-Cotec Pharmaceuticals, China) tablets containing 40 mg of dihydroartemisinin and 320 mg of piperaquine phosphate. A standard weight-based regimen (2.2 mg/kg/day of dihydroartemisinin and 17.8 mg/kg/day of piperaquine phosphate) was administered with milk once daily for 3 days. The fixed combination of DHA-PQ was well tolerated with no serious adverse events reported during follow-up. In this population modeling, nine patients were excluded: two patients did not meet the inclusion criteria, five patients had an incomplete course of medication, one patient self-medicated with other drugs, and one patient withdrew consent. Overall, 45% (109/241) of patients presented with recurrent *P. vivax* malaria during the follow-up period at a median (range) of 56 (14–65) days (**Table 1**).

### Pharmacokinetics of piperaquine

A total of 791 plasma concentrations (435 venous and 356 capillary samples) from 116 patients were quantified and used in the pharmacokinetic modeling. Venous and capillary plasma concentrations were transformed into their natural logarithms and modeled simultaneously using nonlinear mixed-effects modeling. Substantial differences in matched venous and capillary blood concentrations have been reported in a previous clinical study.^17^ A proportional venous-capillary transformation factor was, therefore, used to compensate for the difference between sampling matrices, resulting in 41% higher capillary concentrations compared with venous concentrations.

**Figure 1:**
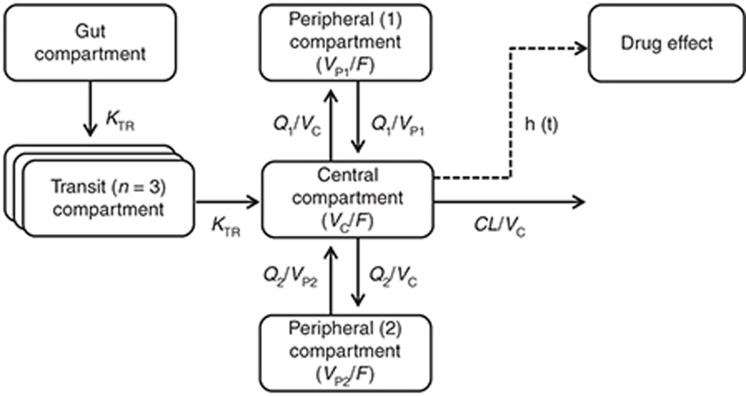
Final piperaquine population pharmacokinetic-pharmacodynamic model in patients with *P. vivax* malaria. *K*_TR_ is the transit absorption rate constant (*K*_TR_ = (*n* + 1)/mean absorption transit time), *CL* is the elimination clearance, *V*_C_ is the apparent central volume of distribution, *V*_P_ is the apparent peripheral volume of distribution, *Q* is the intercompartment clearance, and *F* is the relative oral bioavailability.

The initial structural base models were parameterized as elimination clearance (*CL*/*F*), intercompartment clearance(s) (*Q*/*F*), apparent volume of distribution of the central compartment (*V*_C_/*F*), apparent volume of distribution of the peripheral compartment(s) (*V*_P_/*F*), and absorption rate constant. Several absorption, distribution, and covariate models were fitted to the data to construct the best performing model. Piperaquine pharmacokinetics in patients with *P. vivax* malaria were best described by a three-compartment distribution model (**Figure 1**). A three-compartment disposition model resulted in a significant improvement in model fit compared with a two-compartment model (difference in objective function value (ΔOFV) of 108) with no additional benefit of an additional peripheral compartment (ΔOFV = 0.57). A transit-compartment model with a fixed number of transit compartments (*n* = 3, transit rate constant was set to be identical to absorption rate constant) described the absorption phase better than all other absorption models (ΔOFV > 54.2). A relative bioavailability parameter (i.e., fixed to 100% for the population) was implemented to allow quantification of the interindividual variability in the absorption of piperaquine and resulted in a significant improvement in the model fit (ΔOFV = 81.8).

Body weight, incorporated as a fixed allometric function, improved the model fit significantly (ΔOFV = 31.0) and resulted in better precision and decreased interindividual variability in clearance and volume parameters. Body temperature on admission on *V*_P2_/*F* (ΔOFV = 11.5) and age on *Q*_1_/*F* (ΔOFV = 5.46) were selected in the forward covariate selection but could not be retained in the backward deletion. The estimated interindividual variability was small for the transformation factor, *CL*/*F*, *Q*_2_/*F*, and *V*_C_/*F*, and could, therefore, be removed with no significant impact in the final model (ΔOFV = 2.40). Final pharmacokinetic parameter estimates and secondary parameter estimates are reported in **Tables 2** and **3**.

**Figure 2:**
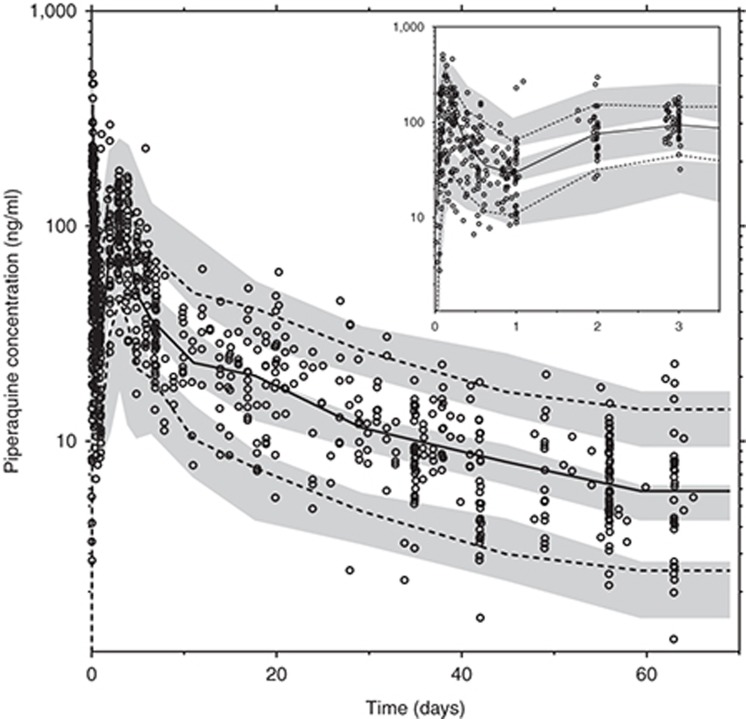
Prediction-corrected visual predictive check of the final model describing piperaquine pharmacokinetics in *P. vivax* malaria. Open circles represent the observed piperaquine concentrations. Solid black line represents the 50th percentile of the observations, and dash lines represent the 5th and 95th percentiles of the observations. Gray areas represent the 95% confidence intervals of the simulated 5th, 50th, and 95th percentiles from 1,000 simulations. The inset shows a prediction-corrected visual predictive check for the first 3 days of piperaquine treatment.

The mean parameter estimates from the simultaneous model with both venous and capillary data were also comparable with estimates when capillary and venous data were modeled separately. The final pharmacokinetic model displayed satisfactory goodness-of-fit diagnostics (**Supplementary Figure S1** online) with a small epsilon-shrinkage of 15.0%. However, a relatively high η-shrinkage (up to 43.5%) could be seen for certain parameters in the final model because of the sparse data. The numerical and prediction-corrected visual predictive checks resulted in 4.6% (95% CI: 2.7–8.1%) and 3.7% (95% CI: 2.5–8.2%) of piperaquine observations below and above the simulated 90% prediction interval, respectively (**Figure 2**).

### Pharmacodynamics of piperaquine

**Figure 3:**
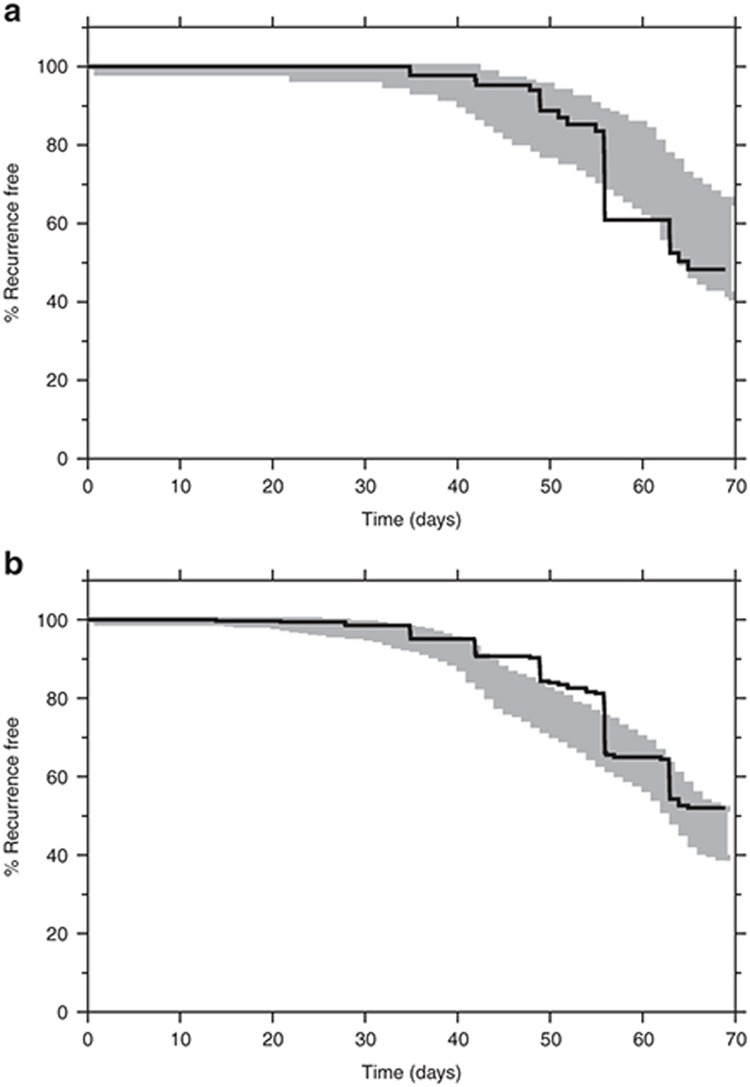
Visual predictive check of the final time-to-event model describing piperaquine pharmacodynamics in *P. vivax* malaria. Gray area represents the 95% prediction interval of the time to recurrent infections from 500 simulations. Solid line represents the observed Kaplan–Meier plot for (**a**) patients in the pharmacokinetic and pharmacodynamic cohort (62 patients, **Table 1**) and (**b**) all patients in the efficacy study (241 patients, **Table 1**).

Piperaquine inhibits asexual parasite multiplication but has no effect on liver-stage parasites. The influence of piperaquine exposure on the risk of recurrent *P. vivax* malaria was investigated in 62 patients for whom both pharmacokinetic and pharmacodynamic data were available. However, efficacy data were available from all the 241 patients included in the study. Pharmacodynamic parameters were evaluated using an interval-censoring time-to-event model,^18^ implemented with the Laplacian estimation method. The pharmacokinetic parameter and variability estimates were fixed to that of the final pharmacokinetic model and used in the pharmacokinetic-pharmacodynamic model. *P. vivax* malaria in tropical regions commonly displays frequent relapses which emerge at three-week intervals when the infections are treated with rapidly eliminated antimalarials.^19^ A multiple surge function was, therefore, implemented to characterize this periodicity in the risk of recurrent malaria. The constant hazard function with a periodically increased risk of relapses described the data well. Piperaquine had a significant (ΔOFV = 20.3) protective effect on the risk of recurrent malaria infections with a required *in vivo* venous piperaquine concentration of 6.92 ng/ml for a 50% relative reduction in baseline hazard (PC_50_). Sex was selected as a significant covariate (*P* < 0.05) on baseline hazard in the forward selection step, but it was removed during the backward elimination resulting in a covariate-free pharmacodynamic model. Final pharmacodynamic parameters were well estimated with high precision (**Table 2**) and simulation-based diagnostics showed good agreement between simulated and observed recurrent malaria infections (**Figure 3**).

**Figure 4:**
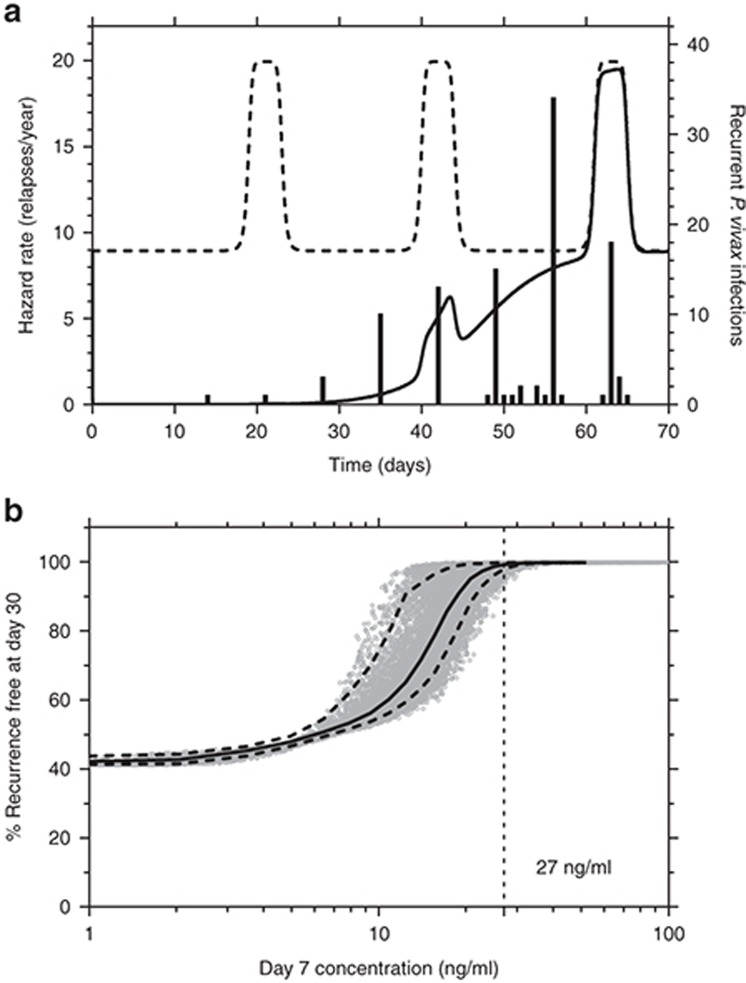
Relationship between piperaquine treatment and risk of relapsing *P. vivax* malaria infections. (**a**) Mean hazard rate of relapsing *P. vivax* malaria versus time in the presence (solid line) and absence (dashed line) of a standard piperaquine treatment (primary *y*-axis). The frequency of observed recurrent *P. vivax* infections for all patients in the efficacy study (241 patients, **Table 1**) is displayed on the secondary *y*-axis. The hazard of the first relapse (3 weeks) is completely suppressed and the hazard of the second relapse (6 weeks) is almost completely suppressed after piperaquine treatment. The model-predicted hazard rate after piperaquine treatment is in agreement with the observed frequency of observed recurrent *P. vivax* infections. (**b**) Simulated day-7 venous piperaquine plasma concentrations and chance of remaining malaria free for 30 days. Open gray circles represent simulated patients. Solid black line represents the 50th percentile and dash lines represent the 5th and 95th percentiles of the prediction interval. The dashed vertical line indicates the predicted day-7 concentration that results in 99% chance of remaining malaria free for 30 days for a typical patient.

The individual patient parasite burden (**Table 1**) corresponded approximately to a total of 10^11^ parasites in a typical adult at admission. This assumes that *P. vivax* does not sequester substantially. DHA-PQ is administered for 3 consecutive days, covering two asexual cycles. Assuming a parasiticidal effect (parasite reduction ratio) of a 10,000-fold parasite reduction per cycle by dihydroartemisinin (without any additional effect by piperaquine) results in a 100,000,000-fold reduction in total parasite biomass during the first two cycles.^20^ At an initial parasite burden of 10^11^ parasites, this would leave ~1,000 parasites to be eliminated by residual piperaquine concentrations to prevent recrudescent malaria. Piperaquine alone has not been adequately assessed in *P. vivax* malaria, but if it is similar to chloroquine, it should provide parasite reduction ratios of ~100 to 1,000 per cycle, so most patients should be parasite free 6–8 days post-treatment if piperaquine levels are sufficient to sustain a maximum parasiticidal effect for this time. If the parasite burden is not eliminated completely, then these remaining parasites can multiply and cause a recrudescent infection. However, a high cure rate of the asexual infection is likely in this study as concentrations of piperaquine after the second asexual cycle (i.e., ≥ 4 days) are highly likely to be able to eliminate ~1,000 parasites. In the first relapse of tropical *P. vivax* malaria (unaffected by drugs and assuming 1–10 schizonts and a subsequent parasite multiplication rate of ~10 per cycle), the hypnozoite-derived hepatic schizonts must liberate 13–17 days after the onset of the primary illness to produce a symptomatic infection at day 21. However, if residual piperaquine concentrations are sufficiently high, they could suppress and eliminate the asexual parasites derived from 10,000 to 100,000 released merozoites and thereby prevent the first relapse completely. If residual piperaquine concentrations are not sufficiently high for complete elimination, they could cause an initial suppression, resulting in a delayed symptomatic first relapse. Only 14% (15 out of 109) of recurrent *P. vivax* infections occurred before day 39, which suggests that DHA-PQ treatment prevents a large proportion of the first relapses (**Figure 4**). The majority of infections (64%, 70 out of 109) occurred between day 42 and 56, suggesting that these are mainly delayed second relapses (**Figure 4**). There will be some contribution from reinfections, but as entomological inoculation rates are probably well below 0.5/person/year for *P. vivax* in this area, this contribution is likely to be small.

## Discussion

The fixed combination of DHA-PQ is a highly efficacious antimalarial drug with an excellent safety profile. Antimalarial treatment with DHA-PQ resulted in lower cumulative risk (54.9%) of recurrent *P. vivax* malaria compared with chloroquine (79.1%) in Thailand.^16^ This difference could be a result of the very potent artemisinin derivative which generally has a rapid onset of action against *P. vivax* malaria,^21^ but it is likely to result mainly from increasing chloroquine resistance and hence the superiority of piperaquine. In fully sensitive *P. vivax* malaria, recrudescence rates are close to zero with chloroquine treatment. Previous studies in this region have shown high relapse rates of *P. vivax* within one month of treatment with a short acting artemisinin derivative.^19^ Thus, both slowly eliminated drugs prevent the first relapse. Whether the lower cumulative risk of recurrent *P. vivax* malaria, following DHA-PQ, results from a higher cure rate of the primary infection or a more effective suppression of the first or the second relapse has been unclear. As relapses can arise from parasites which are either genetically similar or genetically distinct to those causing the primary infection, it is not possible to distinguish with certainty between relapse, recrudescence, or reinfection in patients with *P. vivax* malaria who remain in the endemic area. Recrudescence can occur many weeks after treatment with slowly eliminated drugs, but the pharmacokinetic-pharmacodynamic data modeled in this study suggest that only a negligible proportion (<2%) of the recurrent *P. vivax* infections could be recrudescent.

### Pharmacokinetics of piperaquine

The population pharmacokinetic properties of piperaquine have been described previously in patients with uncomplicated *P. falciparum* malaria.^15,22,23,24^ This is the first study reporting the population pharmacokinetics of piperaquine in patients with *P. vivax* malaria.

The structural three-compartment model described in this study was similar to that reported recently in young children and in pregnant and nonpregnant women with *P. falciparum* malaria.^15,22,25^ Simplified two-compartment structural models in children and adult patients have also been reported reflecting differences in follow-period and/or sampling strategies.^23,24^ A transit-compartment absorption model allowed more flexibility in the absorption phase and provided a clear advantage over other absorption models, although there were not enough data in the absorption phase to estimate both *K*_TR_ and *K*_A_ separately. Large interindividual variability was seen in the absorption of piperaquine as in previous studies.^15,22,25^ Data collected here were too sparse to allow an estimation of the variability between dose occasions, which is likely to have inflated the inter-individual variability. Indeed interindividual variability was high in this study with values up to 138%. Venous and capillary plasma concentrations were successfully modeled simultaneously with a transformation factor of 1.41 (relative standard error of 2.11%). This factor was similar to that from a linear regression between observed venous and capillary plasma concentrations (slope = 1.29), which further supports the appropriateness of the simultaneous model. However, the exact mechanism of this matrix-dependent difference cannot be elucidated from the data collected in this study. The advantage of a simultaneous modeling approach is that the model can be used to simulate drug exposures from any sampling technique and therefore enables literature comparisons.

Body weight was the only significant covariate in this model. This has also been reported in previous studies^15,24^ and was not unexpected, considering the strong biological prior of body weight as a covariate on pharmacokinetic disposition and elimination parameters.^26^ All pharmacokinetic parameters were estimated with good precision (relative standard error < 20%) and the simulation-based diagnostics indicated excellent predictive performance of the final pharmacokinetic model.

Piperaquine *CL*/*F* in this *P. vivax* study was 1.08 l/h/kg, total volume of distribution (*V*_D_/*F*) was 802 l/kg, and *t*_1/2_ was 28.8 days, which were comparable with previously published reports in patients with an uncomplicated *P. falciparum* malaria (mean (range) *CL*/*F* of 1.18 (0.90–1.4) l/h/kg, *V*_D_/*F* of 789 (574–877) l/kg, and *t*_1/2_ of 23.7 (18–28) days).^22,23,24,27,28^

### Pharmacodynamics of piperaquine

The observed high relapse rate of *P. vivax* malaria was expected as DHA-PQ eliminates only blood-stage parasites and does not provide cure of the latent liver-stage parasites. It was expected that piperaquine would delay the time to relapse, as does chloroquine, and this was supported by the pharmacodynamic results. Tropical frequent relapsing strains of *P. vivax* typically relapse at 3–4-week intervals, and the median time to relapse approximates three weeks (21 days) in Thailand after treatment with rapidly eliminated antimalarial drugs (such as artesunate, quinine, and halofantrine).^19^ In an area of much higher transmission in Papua, Indonesia, there were significantly more early recurrences of *P. vivax* malaria following artemether-lumefantrine treatment of *P. vivax* malaria compared with DHA-PQ (cumulative risk of 38 vs. 10% at day 42)^13^ reflecting the failure of the more rapidly eliminated lumefantrine to suppress the first relapse. However, with longer follow-up in a high transmission area, the cumulative reinfection rates converge as everyone is reinfected eventually. A critical and unanswered question has been whether the slowly eliminated drugs prevented, or simply delayed, the relapse. Explicitly, prevention would mean that the relapse emerged but was eliminated by the residual drug levels, and thus, the first observed recurrence is in fact the second relapse. Delay would mean that the residual drug levels reduced multiplication of the relapse, so it reached patency after a substantial delay. In the former explanation, overall relapse numbers might be reduced as this would exhaust the liver burden of hypnozoites more rapidly. A pharmacometric time-to-event approach explaining relapsing *P. vivax* malaria, first presented for amodiaquine treatment in pregnant women,^29^ described the data observed in this study well. An interval-censoring time-to-event model was successfully used since the exact times of relapses were unknown. The risk of relapsing *P. vivax* malaria in patients was explained by a constant baseline hazard (i.e., 8.94 relapses/year) with a 123% increased risk every third week. The pharmacodynamic model was significantly improved by adding a protective drug effect, indicating that piperaquine cured the primary blood stage *P. vivax* infection and fully suppressed a large proportion of the first relapses that would have occurred three weeks after the initial infection. The pharmacodynamic model also indicated that the observed high recurrence rate is likely to be explained mainly by delayed second relapses, although it cannot be excluded that some recurrences are reinfections. This can only be mechanistically evaluated by an individual parasite-data-driven pharmacokinetic-pharmacodynamic model. Simulations using the final pharmacokinetic-pharmacodynamic model (**Figure 4**) demonstrated that minimum day-7 piperaquine venous and capillary concentrations of 27 and 38 ng/ml, respectively, were needed to suppress the risk of relapse for 30 days (i.e., for 99% of simulated patients). This is in close agreement with a previously defined day-7 threshold venous concentration of 30 ng/ml in patients with *P. falciparum* malaria to minimize the risk of recrudescent infections.^30^

In conclusion, the population pharmacokinetic properties of piperaquine in patients with *P. vivax* malaria were described successfully by modeling venous and capillary plasma concentrations simultaneously. Body weight was the only significant covariate and resulted in increasing clearance and volumes with increasing body weight. Times to recurrent *P. vivax* infections were successfully modeled with a time-to-event approach and resulted in a significantly delayed time to recurrent infections during the post-treatment phase of piperaquine treatment. This was explained by prevention of the first *P. vivax* malaria relapse by residual piperaquine concentrations. Piperaquine is a good candidate for the treatment of *P. vivax* malaria and the modeling conducted here demonstrated the added benefit of reduced morbidity compared with other more rapidly eliminated antimalarial drugs.

## Methods

### Ethics approval.

Ethics approval was granted by the Faculty of Tropical Medicine Ethics Committee, Mahidol University, Bangkok, Thailand, and the Oxford Tropical Research Ethics Committee, Oxford University, UK. This study was registered in the ISRCTN Register (ISRCTN87827353). Details on the clinical study design and outcomes have been presented in full elsewhere.^16^

### Drug regimen and blood collection.

All patients received a standard regimen of DHA-PQ. Patients who vomited the dose were excluded from the pharmacokinetic study. A full or half replacement dose was administered if vomiting occurred within 30 min of or between 30 min to 1 h after administration, respectively.

Venous blood samples (3 ml) and capillary blood samples (200 μl) were drawn from each patient for piperaquine plasma measurements. Samples were collected randomly over 69 days (4–7 venous samples and 1–11 capillary samples) from 62 patients and an additional venous sample (5 ml) was drawn from these patients at the time of recurrent *P. vivax* malaria. This random sampling allowed of an adequate coverage of the entire concentration–time profile. An additional 54 patients provided a single plasma sample only at the time of recurrent malaria. Patients were excluded from the study after recurrent malaria. Plasma samples were shipped on dry ice to the Department of Clinical Pharmacology, Mahidol-Oxford Tropical Medicine Research Unit, Thailand, for drug quantification.^31^

### Population modeling.

Piperaquine pharmacokinetics and pharmacodynamics were evaluated using nonlinear mixed-effects modeling (NONMEM version VII; Icon Development Solutions, Ellicott City, MD). Postprocessing and automation were performed using Pearl-Speaks-NONMEM version 3.5.3, Census version 1.2b2,^32^ and Xpose version 4.0^33^ in the programming language R version 2.13.2 (The R Foundation for Statistical Computing). Measurements below the lower limit of quantification were less than 0.5% of the total data and therefore omitted. OFV, calculated by NONMEM as minus twice the log-likelihood up to constant, was used for model selection during the model-building process. A difference in the OFV (ΔOFV) of >3.84 was considered significant (*P* < 0.05) when comparing two nested models with 1 degree of freedom difference. Full details of the pharmacokinetic and pharmacodynamic modeling methodology can be found in supplementary material.

## Author Contributions

J.T., P.T., N.P.J.D., and N.J.W. wrote the manuscript. A.P.P., K.M.L., E.A.A., N.P.J.D., F.N., and N.J.W. designed the research. J.T., A.P.P., K.M.L., E.A.A., and F.N. performed the research. J.T., P.T., and W.H. analyzed the data.

## Conflict of Interest

The Wellcome Trust is a UK-based charity that supports medical research and is independent of any drug companies. It has no financial links with the manufacturers of either the diagnostic tests or the drugs used in this study. The authors declared no conflict of interest.

## Study Highlights


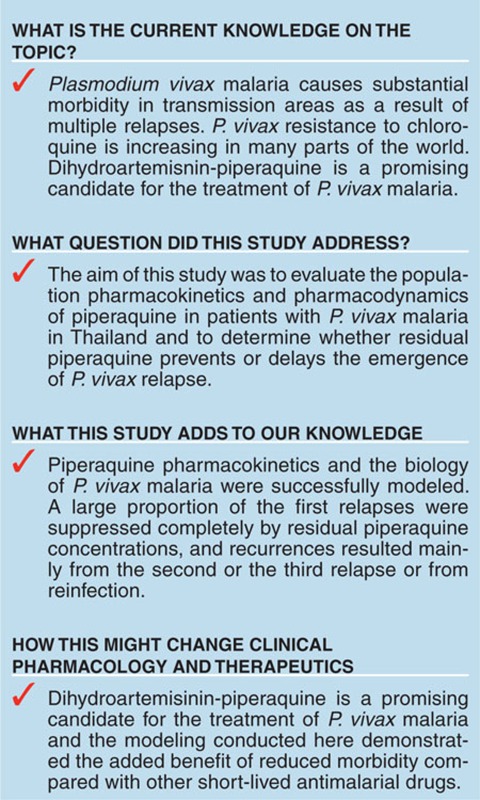


## Figures and Tables

**Table 1:**
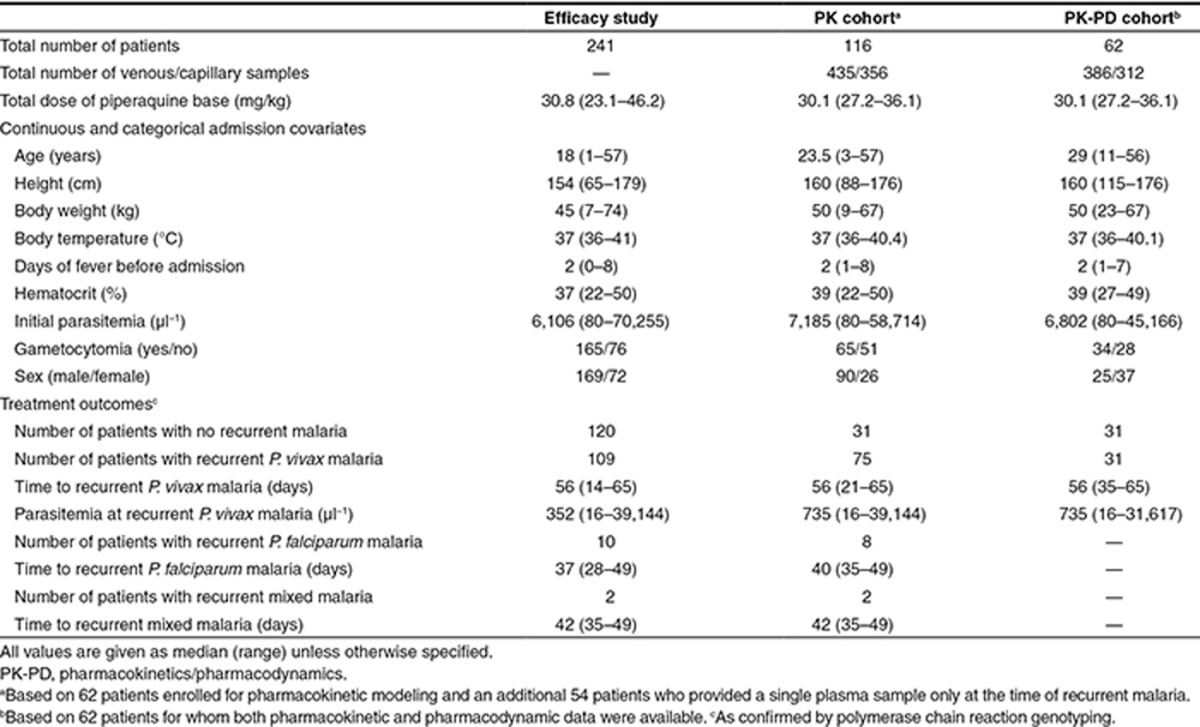
Clinical outcomes and patient demographics of patients with *Plasmodium vivax* malaria enrolled in this study

**Table 2:**
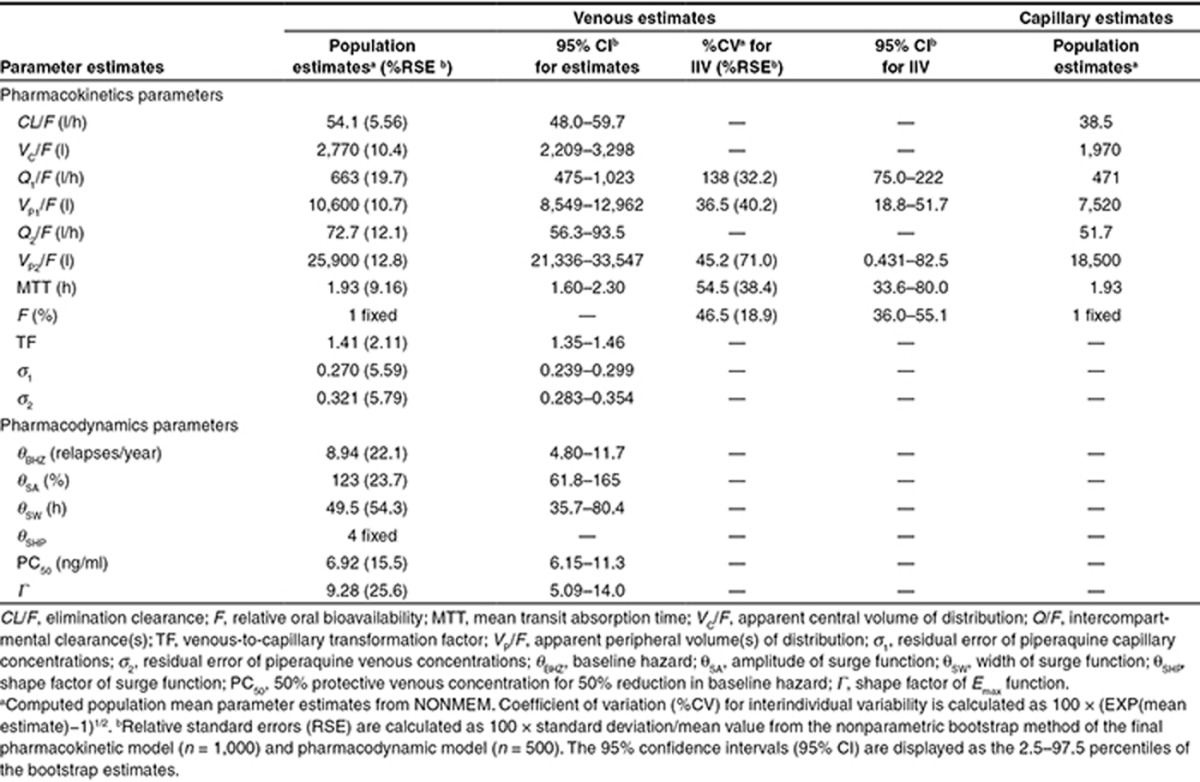
Population pharmacokinetic-pharmacodynamic parameters of the final model describing piperaquine in patients with *Plasmodium vivax* malaria

**Table 3:**
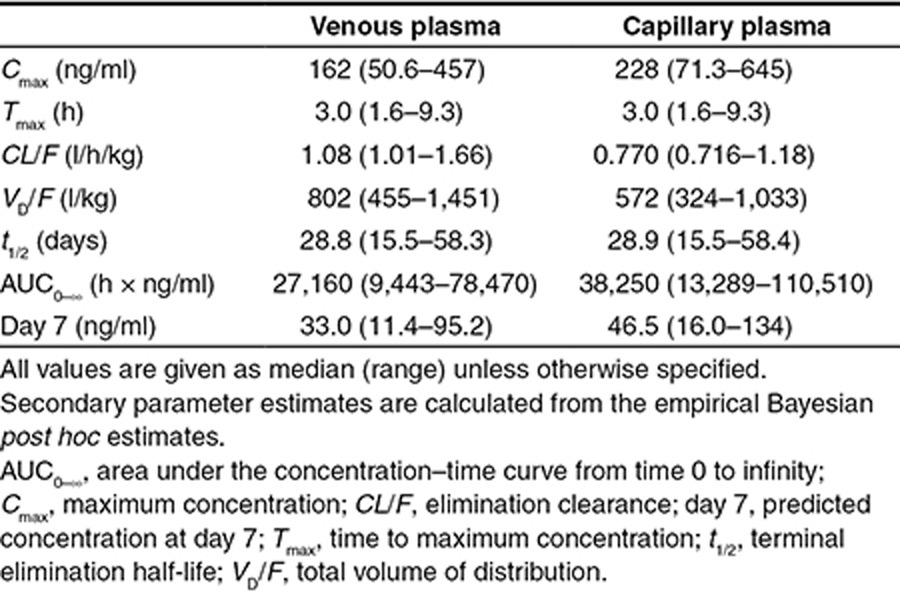
Secondary parameters of the final piperaquine model in patients with *Plasmodium vivax* malaria
