# Supplementary material for: Population Pharmacokinetics and Antimalarial Pharmacodynamics of Piperaquine in Patients With Plasmodium vivax Malaria in Thailand
Source: CPT Pharmacometrics Syst Pharmacol. 2014 Aug 27;3(8):e132–. doi: 10.1038/psp.2014.29 (PMC4150927; doi:10.1038/psp.2014.29)
Supplement: Supplementary Material [file psp201429x2.zip › PSP-2014-0037-T-s01.doc]

**Supplementary material**

**Inclusion Criteria and blood sampling**

The inclusion criteria were males and females aged >12 months, body weight ≥5 kg, *P. vivax* mono-infection (parasitaemia ≥5 per 500 WBC), fever or history of fever (axillary temperature ≥37.5 °C). Patients with hypersensitivity to DHA-PQ, pregnancy or lactation, mefloquine treatment in the previous 60 days, and/or DHA-PQ treatment in the previous 3 months were excluded from the study.

All patients contributed capillary blood sampling for malaria thick and thin films, hematocrit, PCR genotyping, and G6PD deficiency testing. Malaria blood films were stained with Giemsa and parasite count expressed as the number of parasites per 1,000 RBC or per 500 WBC if the parasitaemia was <1% per RBC.

Pharmacokinetic blood samples were centrifuged at 3,000 g (15-24°C) for 15 minutes to obtain plasma and shipped on dry ice to the Department of Clinical Pharmacology, Mahidol-Oxford Tropical Medicine Research Unit (MORU), Thailand for drug quantification. The Department of Clinical Pharmacology is participating in the World Wide Antimalarial Resistance Network (WWARN) quality control and assurance proficiency testing programme (http://www.wwarn.org/toolkit/qaqc).

**Drug assay**

Piperaquine plasma concentrations were measured using solid phase extraction and liquid chromatography coupled with tandem mass spectrometry (1). Triplicates of quality control samples at 4.50, 20.0, and 400 ng/ml were analyzed within each batch to ensure accuracy and precision of the drug assay. The relative standard deviations (RSE) at low, middle, and high concentrations were 4.47%, 2.24%, 1.55%, respectively, for venous samples and 5.16%, 3.15%, 1.71%, respectively, for capillary samples. The lower limit of quantification for both venous and capillary plasma was set to 1.50 ng/ml.

**Pharmacokinetic modeling**

Venous and capillary plasma concentrations were transformed into their natural logarithms and modelled simultaneously using nonlinear mixed-effects modelling. The distribution characteristics of piperaquine were evaluated with 1-, 2-, 3-, and 4-compartment structural models. First-order, zero-order, parallel first-order, mixed first- and zero-order, sequential zero-and first-order absorption models with and without an absorption lag-time, as well as a more flexible transit-compartment model (2) were tested to describe accurately the absorption phase of piperaquine. Pharmacokinetic parameters were assumed to be log-normally distributed with a normally distributed inter-individual random variability (zero mean and variance ω^2^). Between-dose occasion variability was not investigated due the sparseness of data. Residual random variability, which is the difference between the individual model prediction and the measured observation, was modeled as an additive error (essentially a proportional error on an arithmetic scale).

Eleven admission covariates i.e. age, gender, height, body weight, body temperature, day of fever before admission, hematocrit, initial parasitaemia, and gametocytaemia were tested formally using the final base model. A stepwise covariate addition (p<0.05) and elimination (p<0.01) approach was used to evaluate these potential covariates on all population parameters. Linear, hockey-stick and exponential covariate relationships centered on the median value of the population were evaluated for continuous covariates. Gender and gametocytaemia were evaluated as categorical covariates. Body weight (centered on the median weight of the population) was also evaluated as a fixed allometric function on all clearance and volume parameters (exponent of 0.75 for clearance parameters and 1.0 for volume parameters, respectively) (3).

Model goodness-of-fit was assessed by plotting measured drug concentrations against population predictions and individual predictions and by plotting conditional weighted residuals (4) against population predictions and time. Additional diagnostics and the predictive performance of the final model were evaluated by numerical and prediction-corrected visual predictive checks based on 1,000 simulations (5). Bootstrap diagnostics (n=1,000) were performed to evaluated robustness of the final model and to get RSEs of parameter estimates as well as nonparametric confidence intervals (CI) around these.

**Pharmacodynamic modeling**

The constant baseline hazard (θ_BHZ_) was applied initially to describe the risk of recurrent *P. vivax* malaria [Eq. 1]. The exact time of recurrent malaria patency was not available since patients were screened once weekly for the presence of *P. vivax* parasites. Time of first positive malaria screen was therefore coded as the end of the event-interval and the last visit with a negative malaria screen was coded as the start of the event-interval. Patients with no recurrent malaria were censored at the end of study, at the time of drop-out or at the time of recurrent *P. falciparum* malaria. Thus, the survival describes the probability of not having a *P. vivax* malaria event within a certain time interval and is a function of the cumulative hazard within this interval. The survival up to the event-interval was calculated according to equation 2 [Eq. 2] and the survival during the event-interval was calculated according to equation 3 [Eq. 3].

$H\left(t\right)=\theta_{BHZ}$ [Eq.1]

$S\left(t\right)_{start}=e^{\left[-H\left(t\right)_{start}\right]}$ [Eq.2]

$S\left(t\right)_{event}=1-e^{\left[H\left(t\right)_{start}-H\left(t\right)_{event}\right]}$ [Eq.3]

where S(t)_start_ is the survival and H(t)_start_ is the cumulative hazard up to the start of the event-interval (i.e. time of last negative malaria screen), S(t)_event_ is the survival and H(t)_event_ is the cumulative hazard during this interval. The multiple surge function [Relapse(t)] was implemented as [EQ. 4 & 5].

$Surge\left(t\right)=1+\left[\frac{\theta_{SA}}{\left(\frac{\left(t-\theta_{PT}\right)^{2}}{\theta_{SW}^{2}}\right)^{\theta_{SHP}}+1}\right]$ [Eq.4]

$\mathrm{Re}lapse\left(t\right)=Surge\left(t\right)_{3wk}\times Surge\left(t\right)_{6wk}\times Surge\left(t\right)_{9wk}$ [Eq.5]

where θ_SA_ is the estimated peak amplitude, θ_PT_ is the fixed peak time (i.e. 3 week intervals), θ_SW_ is the estimated half of the peak width at the middle of peak amplitude and θ_SHP_ is the steepness/shape factor of the surge function. Individually predicted venous piperaquine concentrations [C(t)] were related to the hazard via an inhibitory maximum effect (E_MAX_) function [Eq. 6], where PC_50_ is the venous piperaquine concentration required for a 50% relative reduction in baseline hazard and γ is a shape parameter (γ=1 for the E_MAX_ function, γ≠1 for sigmoid E_MAX_ function).

$h\left(t\right)=\theta_{BHZ}\times\mathrm{Re}lapse\left(t\right)\times\left[1-\frac{C\left(t\right)^{\gamma}}{C\left(t\right)^{\gamma}-PC_{50}^{\gamma}}\right]$ [Eq. 6]

The influence of available covariates on the above hazard function was investigated using a stepwise approach, identical to that for the pharmacokinetic covariate modeling described previously. Pharmacodynamic model selection was based on the difference in OFV and simulation-based diagnostics (i.e. observed Kaplan-Meier curves overlaid with the 95% CI of 500 simulated curves) from the 62 patients included in the pharmacokinetic-pharmacodynamic cohort. The simulated Kaplan-Meier curves were also overlaid with observed recurrence from all 116 patients contributing pharmacodynamic data to evaluate the predictability of the developed pharmacokinetic-pharmacodynamic model. Bootstrap diagnostics (n=500) were performed for the final pharmacokinetic-pharmacodynamic model.

**References**

(1)	Lindegardh, N., Annerberg, A., White, N.J. & Day, N.P. Development and validation of a liquid chromatographic-tandem mass spectrometric method for determination of piperaquine in plasma stable isotope labeled internal standard does not always compensate for matrix effects. *Journal of chromatography* **862**, 227-36 (2008).

(2)	Savic, R.M., Jonker, D.M., Kerbusch, T. & Karlsson, M.O. Implementation of a transit compartment model for describing drug absorption in pharmacokinetic studies. *J Pharmacokinet Pharmacodyn* **34**, 711-26 (2007).

(3)	Holford, N.H. A size standard for pharmacokinetics. *Clinical pharmacokinetics* **30**, 329-32 (1996).

(4)	Hooker, A.C., Staatz, C.E. & Karlsson, M.O. Conditional weighted residuals (CWRES): a model diagnostic for the FOCE method. *Pharmaceutical research* **24**, 2187-97 (2007).

(5)	Bergstrand, M., Hooker, A.C., Wallin, J.E. & Karlsson, M.O. Prediction-Corrected Visual Predictive Checks for Diagnosing Nonlinear Mixed-Effects Models. *The AAPS journal* **13**, 143-51 (2011).

**Supplementary Figure Legends**

**Fig S1. Goodness-of-fit diagnostics of the final model describing piperaquine pharmacokinetics in patients with *P. viax* malaria.** Open circles represent the observed piperaquine concentrations. Broken lines represent a locally weighted least-squares regression. Solid lines represent the line of identity. The observed piperaquine concentrations, population predictions, and individual predictions were transformed into their logarithms (base 10).
